# Fatherhood and children with complex healthcare needs: qualitative study of fathering, caring and parenting

**DOI:** 10.1186/1472-6955-10-5

**Published:** 2011-04-15

**Authors:** Lucie Hobson, Jane Noyes

**Affiliations:** 1 National Institute for Social Care and Health Research Clinical Research Centre North Wales Research Network, UK; 2 Centre for Health-Related Research, Bangor University, UK

**Keywords:** Disabled children, complex continuing nursing care, fathers, community nursing, qualitative

## Abstract

**Background:**

Fathers are increasingly providing substantial amounts of technical and nursing care to growing numbers of children with complex healthcare needs. This exploratory study reports some of the first in-depth evidence of fathers' experiences and presents a research agenda in this critically under-researched area.

**Methods:**

We conducted in-depth qualitative interviews with 8 fathers who provided a substantial amount of complex technical and nursing care for their child at home. The aim was to describe their experiences of fathering, parenting and caring. Interviews were recorded, transcribed and analysed using Burnard's approach, which has commonalities with phenomenological and content analysis.

**Results and Discussion:**

Fathers enjoyed their caring role and found it rewarding and at times stressful. They instituted structured regimes, which focused on the father/child/family. Performing intimate care posed specific challenges for which there is no guidance. Children's community nursing was highly valued. Fathers generally rejected the need for specific father-focussed services, as such provision would induce guilt feelings. Fathers reported positive relationships with their children and partners.

**Conclusions:**

Key areas for future exploration include gaining a better understanding of fathers' motivations and styles of caring, developing interventions to support fathers' caring role, developing guidance on intimate care, and delivering tailored services to fathers in a family context. There is little understanding of fathering and caring by non-resident, teenage and step-fathers. Finally, knowing more about resilience and coping of fathers in strong relationships with partners and children may help inform interventions to support fathers who do not feel able to stay with their family.

## Background

Over the last two decades there have been steady increases in the number of children with complex health needs cared for at home [1]. Children with complex health needs include those requiring tracheostomies, long-term ventilation, assisted enteral or parenteral feeding, administration of intravenous drugs, and may include children with severe movement disorders or mobility impairments, some children also have sensory impairments [2].

It is now common for daily complex nursing care required by these children, sometimes involving highly technical medical and nursing procedures, to be carried out at home by parents [3]. Health and social care services and specifically children's community nursing (CCN) teams have been faced with the need for rapid adaptation to support increasing numbers of children with complex health needs [4].

In recent years, children's health policy has increasingly recognised changing family demographics and roles of fathers in caring for their children. For example, the Children's National Service Framework (NSF) in England [5] identifies that fathers play an integral role in the family when a child is disabled or has complex health needs.

### What is known about fathers and complex caring?

There is a relatively small literature that currently informs children's community nursing practice relating to fathers who provide complex nursing care at home. A number of studies found that some fathers want to take an active role and share their child's care [6-8], while other fathers found taking an active role difficult, challenging and stressful [9-11].

Fathers' employment and responsibility to remain the provider for the family restricts time to care, resulting in mothers becoming primary caregivers [8,9,12]. Some fathers reported a gradual change in their role from breadwinner to care provider [6]. In two studies fathers found that employment was paramount in providing an opportunity to escape from the situation and their caring role [8,10]. Whereas, several fathers in a study by Sullivan-Bolyai [7] felt that their role was to provide respite for their partners when not at work.

A study examining the experiences of fathers of children with cancer found that several fathers felt they lacked experience in their child's condition as mothers wanted to carry out all of their child's care [9]. Fathers also felt that they had to appear strong and able to cope with the situation as this was expected [11].

When parents were interviewed together it was generally found that they were performing multiple roles in caring for their child including being a parent and providing skilled nursing care in addition to organising services and advocating for the child [3,12-14].

Published literature was helpful in providing confirmatory contextual evidence, but inadequate in describing and analysing in any depth the range and scope of roles that fathers now engage in at home when caring for children for complex needs. In particular, we found little research with fathers who care for their children full-time or who provide a significant amount of complex nursing care. We therefore decided to undertake an exploratory qualitative in-depth study with fathers who provide full-time or a significant amount of their child's complex nursing care. This study sits within a larger programme of health services research focussing on disabled children and those with complex healthcare needs.

## Methods

The aim of this exploratory qualitative study was to describe the experiences of fathers who cared for their children with complex health and nursing care needs. In particular, we wanted to understand better how fathers were managing their children's complex care and the meaning they attached to their roles. We used Burnard's approach [15], which provides a systematic method of analysing interview data by breaking down the text into meaning units, developing a category system, and grouping together ideas of a similar sort. Burnard describes this analytical process as being similar to phenomenological analysis but also considers the approach to have much in common with content analysis. In addition, we adopted a principle from Heideggerian hermeneutical phenomenology [16,17]_, _in that we did not attempt to bracket our knowledge as children's nurses who had extensive experience of working with families, and increasingly fathers, at home.

The objectives of the study were to:

• Describe the roles fathers engage in within the family with respect to the child with complex needs;

• Ascertain what fathers felt about their current roles;

• Explore what informal and formal support fathers currently receive or would like to receive in fulfilling their roles.

### Data collection

Face to face interviews using a broad topic guide recorded fathers' experiences. We used our knowledge of the literature and clinical experience of working with fathers to develop a simple broad topic guide. Open-ended questions and prompts were used throughout to give fathers the opportunity to expand on their own personal experiences. Field notes were recorded to contextualise interviews.

### Participants

A social definition of fatherhood consistent with health policy was adopted which included biological and stepfathers and men that played a significant role in the life of the child [18].

We adopted a purposive sampling strategy and aimed to recruit up to 8 fathers in this initial exploratory study. Fathers were recruited via a CCN team and were not known to the researchers. The CCN team were briefed that we particularly wanted to focus on fathers who took an active role in caring for their child. The CCN team distributed 46 letters of invitation and in most cases followed up in person to explain about the study and what it involved. We recruited the first eight fathers who responded to our initial invitation and confirmed that they provided a significant amount of their child's complex care. We did not need to send additional reminders to encourage sufficient fathers to express an interest in participating. Of these eight fathers, seven were biological fathers and one was a stepfather. Four fathers were full-time carers and four were in full-time employment but heavily involved in their child's complex nursing care.

**Figure 1:**
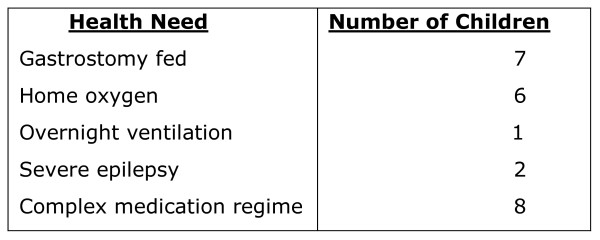
**Health needs of children included in the study**.

The children whose fathers were included in the study were aged from 16 months to 16 years. A number of the children had rare genetic syndromes, which are not outlined here to maintain confidentiality. Children's main health needs which required additional complex care are outlined in Figure 1.

Five of the children attended special school, two attended mainstream school and one attended a pre-school child development centre.

Four of the mothers were full-time mothers and carers and four were employed either full or part-time. Seven of the fathers interviewed had other children. Six families had other siblings living at home while two had older children who had left home.

### Data analysis

Interviews were recorded and transcribed verbatim. Following Burnard's method, after cleaning transcripts we worked on electronic text-based documents. Burnard describes meaning units as a discrete phrase, sentence or series of sentences which conveys one idea or one related set of perceptions. Each transcript was read and re-read by the researchers. The text was highlighted and organised into meaning units to which were attached labels. In an ongoing iterative process, we spent time together developing and refining the meaning units, and an overarching category system that captured all meaning units. We did this by charting and mapping discrete words, sentences and series of sentences until the meaning units were finalised, and then grouped together in a category system that accounted for all meaning units. Burnard (1994 - p114) describes two types of categories: literal categories and descriptive categories. Literal category labels are identified as 'the literal content of interviews, including definitions and raising of issues, such as educational issues'. Whereas Descriptive category labels, are identified as being 'less literal and are more geared towards catching the flavour of what the respondent is saying, such as learning a role or facing a fear'.

**Figure 2:**
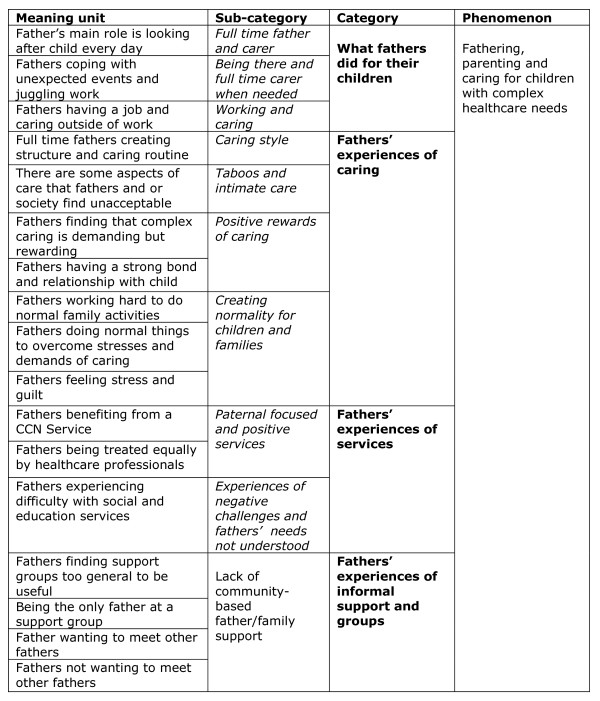
Analytical model of fathers caring for children with complex healthcare needs.

The meaning units and categories are displayed in an analytical model in Figure 2.

### Research Governance

The study was approved by the University and Local Research Ethics Committee and research governance requirements of the relevant organisation were met.

## Results and Discussion

Meaning units, and the category system shown in Figure 2 are discussed in detail below. In our analysis, almost all of the categories can be defined as 'descriptive' in that they capture the flavour of what fathers told us about their roles. We did not identify any 'literal' categories.

We also adopted terminology that is commonly used by mothers and mothers who are carers in the UK to describe their roles. Mothers who are not employed outside of the home commonly describe themselves as 'full-time mothers' or in the case of mothers who provide nursing care to their children are commonly referred to as 'full time mothers and carers'.

We have therefore adopted similar terminology and refer to 'full-time fathers' as those who provide nursing and care full-time as their main role within the family, and 'non full-time' or 'part-time' fathers as those who provide a substantial amount of nursing and care for their child for some but not all of the time.

### What fathers did for their children

#### Full-time father and carer

The experiences of four full-time fathers provide evidence of a planned shift in their roles from mainly breadwinner to main care provider. Three families made the decision for fathers to stay at home because of the child's need for full-time complex care. Father 6 explained that he was due to stay at home before they were aware of his child's difficulties. He felt that he had not had this opportunity with his older children. Father 6 had planned to return to work when the child was aged around two but had now decided to remain home as long as needed. Father 7 initially returned to work but his daughter's condition deteriorated and he felt his priorities were at home. He at times missed work but could not cope with both at that time.

#### Being there and full-time carer when needed

When exploring arrangements for hospital admissions and appointments all fathers interviewed wanted to attend hospital appointments with their child. When a child was admitted either for a planned or emergency stay non full-time fathers tended to step in to provide full-time care for the siblings at home while mothers mainly stayed with the child at hospital. A number of non full-time fathers also stayed with the ill child where possible to provide care. Father 8 explained the importance of attending hospital appointments:

'We go to (specialist hospital) and we come out and we go and watch the pictures on the way back and have a pizza or something as a little treat, so we try and have a bit of family bonding time as well.'

#### Working and caring

The fathers who were in employment openly discussed how they thought primarily working and earning was the father's role. Similar to Gravelle [12], employment concerns and constraints affected the level of care working fathers provided, but they all provided complex nursing care for their child. Father 1 described his role as:

'Obviously I'm not here some of the time because I go to work and earn the money and sort of bring the food in and keep a roof over our head'.

In contrast to other accounts, father 1 thought that all fathers were the breadwinner. He did not appear to consider other options of caring roles.

Father 2, who was also in full-time employment and worked away from the family home during the week, explained how his role was to support the child's mother:

'I am conscious when I return home from work at the weekends that I do not interfere and disrupt the routine that works so well when I am away. My main aim is to support my wife'.

Father 8, who was in full-time employment, experienced a number of issues with his employer and was questioned about taking time off work to be involved in his child's care and hospital treatment.

Father 8 explained that he said to his boss:

'I can't make this particular day and when I explained he said.. Well what's your wife doing? and I sort of looked at him and he said... And who's the main breadwinner?'

Father 8 wanted to take an active part in his child's care whenever possible, but this became increasingly difficult when attempting to negotiate and justify time away from work.

Understanding decision-making about who does what in the family and in particular which family members deliver complex and technical nursing care to children, and how these decisions are negotiated are of interest from a nursing perspective. We particularly wanted to find out about the motivations of fathers choosing to give complex technical medical and nursing care. From a nursing perspective both male and female nurses deliver this type of care so the nursing role of delivering complex care to children is not typified by gender although there are more female nurses than male nurses. Interestingly we know from our clinical experience that child safeguarding and dignity of care policies in many health organisations in the UK frequently require male nurses to work with a chaperone when performing intimate care on children. In the context of this study, we explored the substitution of professional nursing care that could be delivered by male or female nurses, to fathers. In the UK generally, with traditional 'male' jobs rapidly disappearing there has been a longstanding shift towards fathers taking more routine and active roles in sharing the parenting of their children. All the self-selecting fathers who volunteered to participate in this study had chosen to extend their already active parenting roles and were not fazed by operating and managing complex technology or delivering complex procedures or treatments that required a high level of skill and training. Indeed, many of them equated delivering this level of technical care as their unpaid job.

### Fathers' experiences of caring

#### Caring style

Routine was talked about by most of the fathers. Father 5 who was a full-time carer explained that keeping a strict daily routine made the situation easier to cope with. Whereas, Father 3 found it difficult to set a routine whilst his child was awaiting an educational placement so he planned to structure a routine when this was organised.

The structure and implementation of routines made by the fathers had characteristics that appeared to be different from our experiences of working with mothers. A structure and routine associated with paid work had been replaced by a structure and routine of unpaid work in the form of caring. For example, fathers structured the routine around the father-child dyad when other family members were not around and did not tend to integrate a social function such as meeting adult friends/neighbours in social contexts.

For some families, the level and intensity of care required by their child meant that opportunities for social lives outside of the home were greatly reduced. Some children required 24-hour care seven days a week. When unwell, children often required complex medical and nursing procedures to be performed during the night. Parents were carrying out these tasks at night and continually missing periods of sleep. Similar to Kirk et al's work [3], fathers found it difficult to distinguish their parenting and technical caring on what could be seen as a long and consuming journey with their child as they progressed along their illness trajectory [19]. Father 7 highlighted part of his role as follows:

'.. doing medications and setting pumps up is nothing but it's totally different when you're giving IVs [intravenous drugs]... because I can go to bed early whereas when she's on IVs I can't go till 12 till after 12 at night because at 12 is when she'd have the last one'.

For this father the unpredictable need to increase vigilance and intensity of technical nursing care in response to acute illness was a regular occurrence due to the complexity of the child's condition and needs. He was frequently required to meet his child's nursing needs before engaging in basic parenting and nurturing of his child.

Father 7 went on to explain that he would rather care for his daughter at home (rather than hospital) but it could be difficult when she required additional treatments.

Two children requiring overnight feeds or oxygen slept in their parent's bedroom. Their fathers explained that this was the only way they could manage the situation and gain a little rest even though it was disrupted throughout the night. Heaton et al [20], have described previously the sleep disruption experienced by families providing complex and technical care at home and the limited opportunity for short breaks.

#### Taboos and intimate personal care

Providing intimate care to children and young people with complex needs was inevitable for families both at home and in public places. Intimate personal care posed particular challenges (and sometimes taboos) for fathers, and specific challenges for both fathers and families as children reached puberty - for example when girls started menstruating and needed support to change sanitary products, and maintain cleanliness and comfort. Other procedures such as catheterisation and cleaning intimate bodily places caused anxiety and concern.

During the interviews the fathers provided examples of times they felt uncomfortable providing personal care to their child. The fathers were not specifically stating that they would not deal with these issues. However, it was clear that they were unsure what was appropriate and additional support was required to help them work through their anxieties. They also needed practical help to consider and plan appropriate ways of giving intimate personal care to their children and consider how best to support their children to receive intimate personal care from their fathers.

Issues of privacy, dignity and safeguarding are contextual and bounded by societal norms. The UK is a multi-cultural society, within which there are vastly different cultural, social, and religious preferences concerning the acceptability of receiving personal care from specific people and those of a specific gender. British indigenous culture concerning nudity and mixed sex public facilities is noticeably different than other European cultures such as Germany and Scandinavia. For example, in the latter countries mixed sex changing facilities are not unusual, and nudity is common in mixed sex saunas. Whereas in Britain adult public changing facilities are separate and people always wear swimming costumes in public saunas.

These fathers also give care to their children in a UK society that operates a child safeguarding system that requires mandatory vetting of all adults working with vulnerable people, although fathers are exempt from being vetted if giving personal care to their children. These vetting requirements can also be interpreted as the increasing formalisation and bureaucratisation of care. In the UK, such arrangements were to have been extended to around one third of the adult population who came into contact with children (other than their own) until plans were rescinded by the new UK Conservative/Liberal Democrat coalition government. British society also places clear responsibility on people to report child safeguarding concerns to local social services and those reporting concerns do not have to give their name.

There are guidance and protocols for family support services [21] and in nursing practice there are universal standard guidelines about use of chaperones and the appropriateness of doctor/nurse gender when undertaking intimate examinations or delivering intimate nursing care to children [22,23]. Fathers' anxieties and concerns may well be influenced by practices of nurses working in their homes who follow professional guidance. Such guidance outlines that nurses (especially male nurses) can find themselves in vulnerable positions that may be misinterpreted as inappropriate or abusive by other children and adults (such as performing catheter care, inserting rectal medication and bathing children).

Nurses are encouraged to perform care in community settings with a parent, another carer or a chaperone present so that care can be observed and not misinterpreted. The guidance recognises that there may be occasions where male nurses are working alone and unobserved by colleagues or the child's parents. In these situations, if a chaperone is not available male nurses are advised to move care to a safe observed area. Whilst this guidance is designed to protect nurses from misunderstanding, misinterpretation and accusations of child abuse, the guidance does not always take into account the perspective of the child and their right to privacy, or the practicality or appropriateness of moving care to a place that can be observed by a parent or another responsible adult.

There is little (if any) information for parents, especially fathers, about providing intimate personal and complex nursing care such as intermittent catheterisation for their children at an age when it would no longer be appropriate for parents to do so - especially in public or commercial toilet facilities. For example, there is little (if any) guidance for fathers about taking their daughters with complex healthcare needs into male changing rooms or toilets and undertaking intimate personal care - even if using the male disabled facilities for privacy. Despite provision of separate high quality gender specific disabled facilities, fathers can feel uncomfortable at the prospect of undertaking personal and intimate care for their children in case it is misconstrued as child abuse or inappropriate sexual behaviour. Fathers who were full-time carers who wanted to enjoy an ordinary social life with their children had to find a way of planning and managing these situations, whereas other fathers avoided performing intimate personal care unless they had to as they felt it was inappropriate.

Father 4 who was in full-time employment and helped care for his growing daughter who needed continence care explained:

'I mean she (mother) does the bulk of it (personal care) I tend not to get involved in the personal care unless I absolutely have to and it's not because I don't want to get involved in changing nappies it's just about what is and isn't really appropriate'.

Our study highlights that fathers are generally finding it difficult to interpret what is considered appropriate, and how best to manage their child's personal care without their caring being misinterpreted as abuse by other children or members of the public.

##### Positive rewards from caring

Despite the challenges and demands described previously, there were many positive and rewarding aspects of caring for their child that fathers in this study identified.

Fathers explained that they found their role rewarding irrespective of whether they provided some or all care for their child. Fathers 5, 7 and 8 described the strong bond they had developed with their children, which resulted in them feeling more attached to their child.

Father 3, who due to personal circumstances had spent the previous year at home caring for his child full-time, described the experience as a "golden year".

Father 1 described the positive aspects of caring for his child:

'I think it's being able to do things for her and to see her happy and the reaction you get from her. All of them are best bits'.

Fathers also explained the importance of being a family and involving the child with everything that the family did together. For example, Father 1 described this as:

'It's seeing her being involved and I think the rewarding thing is being able to involve her in everything that everyone else is doing'.

In contrast, the wider literature tends to focus on problematic relationship issues and the high incidence of family breakdown with fathers leaving and losing contact with their children [8,9,11]. This study provides new insights into how fathers developed the necessary technical, nursing and caring skills and remained fully engaged with the family by evolving their roles by either taking on or sharing care. As a consequence of their shared experiences and caring they reported positive outcomes for themselves, their partners and children. We found few other studies, in which overall fathers reported stronger personal relationships with their children and partners as a result of providing shared or total care of their children with complex needs (See for example [9,24]).

##### Creating normality for the child and family

Similar to findings from other studies with predominantly mothers - overall caring was stressful and presented difficult challenges, which fathers strived to overcome to create a 'normal' family life [9-11].

There is evidence in the literature about adjustment and coping with a child's disability and stress experienced by parents and technical caring (see for example, [16,25]). There is, however, little known about the specific stresses experienced by fathers who when in sole charge have to undertake multiple complex technical nursing and caring tasks juggled with usual child care, play and managing the home.

Three fathers described feelings of guilt, high levels of stress and difficulties whilst coping with their situation and trying to maintain normality. One father described how he felt like he was constantly juggling everything. Another father who worked away from home during the week explained that when he was at home he would try and do as much as possible to balance out the guilt he was experiencing.

Father 8 explained how he attempted to keep life as normal as possible:

'When (child) was on his really low and his really bad side, we also tried to keep life going and ticking, you know, we never stopped not going on holiday or we never stopped having a weekend away.'

Father 2 described his attempts to constantly maintain normal family life and include his child in all aspects of life:

'My philosophy on anything is you don't give in on things so I'll always make time and effort to do what I can with him, as much as I can to do for him I will, so the weekend's devoted to (child), there's not much space to do anything else, we try to make sure that we have as normal a life as we can...'

Overall, there was a strong desire amongst fathers to achieve a sense of normality within their family and achieving normality was central to their caring role.

Within the context of caring for a child requiring complex nursing and technical care fathers described how difficult it is to achieve normality and do 'normal' things with children with complex healthcare needs. Just leaving the house took huge organisation and planning to manage and anticipate risks and untoward incidents. The fact that fathers wanted to go the extra mile to achieve a sense of normality is important. These behaviours also demonstrate resilience in the face of adversity and contrast with numerous accounts in the literature of socially excluded and isolated families with disabled and life limited children. Although fathers, children and families also experienced degrees of social exclusion and isolation, they went to extraordinary efforts to enable their children and families to experience ordinary childhood activities, frequently at the expense of their own independent social lives.

Father 3 described how he attempted to maintain a balance within the family and how his relationship had strengthened due to their specific situation:

'To me there is a definite balance that has to be kept and whilst normally there is a relationship with your partner, or your wife, which is very important, and (wife) and I are married, that immediately gets put in the background, that's not important because there's a strength there anyway when a problem comes in'.

A number of the fathers dedicated all of their spare time to caring which resulted in little time with their partner. Also the opportunity to maintain a hobby or sporting activity was also not a priority for the fathers.

In a study of children with cancer, some fathers identified that their relationships were also stronger due to their situation [9]. Father 4, whose child also had severe challenging behaviour as part of his condition, explained how gaining support from, and providing support to, his partner helped to get through the difficult times.

Father 7, who was a full-time carer, said that at times caring could be boring especially when the child is in school. When asked what he did in his spare time he explained that it was difficult to attend anywhere on a regular basis as the child was often unwell and unable to attend school. He therefore needed to be available at all times.

### Fathers' experiences of services

All of the children involved in the study were receiving regular contact from a CCN team, therefore findings have limited generalisability as many families do not receive this type of support due to lack of CCN provision [26].

Fathers described how their CCN liaised between professionals and often acted as a key worker. Three children received respite at home provided by a CCN carer. One child was offered respite at home but the father explained how they had declined the service at present. One child had a joint funded package of respite between the health service and social services. Six out of the eight children attended a children's hospice for respite or short break care on a regular basis.

#### Paternal focused and positive services

Fathers wanted to be recognised for their expertise and were satisfied with health and CCN services they received, and most found the level of support to be high quality. There were subtle differences between fathers' interpretations and perceptions regarding the purpose and focus of supportive services, compared with our more extensive experiences of working with mothers. For example, when asked about services aimed directly at fathers the response was that services already met their needs. Father 2 explained:

'As long as the systems are in place and the care's in place that support the family then I think that fathers would be much more, be comfortable with it, whereas anything that's just for fathers could actually add to the guilt'.

This inverse level of guilt if services are individually tailored to fathers' needs warrants further exploration and consideration in terms of service planning. We were not able to ascertain why fathers felt guilty if interventions and services were focussed specifically on their needs, other than fathers may not want any specific focus on them and may not want to be considered as needing additional State support in order to cope. Fathers were also generally caring in greater isolation than mothers, who in our experience have different and more established social networks and higher motivations to maintain a social network whilst parenting and caring. For mothers who care, there are opportunities to meet other mothers at various mother/child groups, social network sites and at school. For example, there are groups of mothers of disabled children who communicate via Facebook, but we have little evidence as to whether fathers who care are gaining support from similar types of activities. Fathers' needs in terms of managing as a solitary carer with a child-focused caring routine at home, with limited social contact whilst caring, warrant specific attention. There is little evidence in the literature to help understand if certain types of interventions would be acceptable or whether fathers would benefit from interventions to better understand their behaviours and support their engagement in adapting their caring routines to where appropriate incorporate social networking opportunities.

Some fathers said that they did perceive some gender bias in the way they were treated by family members. Father 5 felt that family members were more sympathetic with the child's mother as they assumed she was more upset with the situation than he was, but he did not feel as though he was treated differently by any professionals:

'I feel like people have treated us really equally and I don't think anyone's assumed that (wife)'s more upset than me, and I don't think that people have assumed that (wife) needs to be told more information than me, I think we have been treated pretty equally'.

He explained that he was often equally upset and he was the main carer. Family members, nonetheless, expected him to cope with the situation and often focused their support on the mother.

Like parents in countless other studies, Father 6 felt he would like more information regarding service availability and choices. He explained:

'If we'd been left to our own devices we wouldn't have had access to anything and so we would have probably struggled for a couple of years without the things that (child) needed.'

The support received subsequently by the CCN and CCN service was valued by fathers who described positive and effective relationships with their nurses.

Father 7 outlined the support of their CCN:

'.. We call her because (name of nurse) says we know, we're old hands at it, we know what we're doing so we just call (nurse) if we're concerned or obviously if we need equipment like for medications we just give her a ring or if this you know if she's (child) not looking too well but we don't think we need to go to hospital then we can ring (nurse) and she will come in'.

Father 8 explained how their CCN supports them:

'.. they (CCNs) just say the right things at the right time, if you're a bit low and a bit down, they just say the right thing, and its good'.

The children's hospice provided excellent support and respite to a number of families. Fathers utilised the hospice as part of a whole family or left the child there for care in order to spend time with the rest of the family or child's mother.

Father 5 was grateful for the support from the CCN team. He explained:

'We wouldn't have been able to come home without (nurse) definitely, and at first when we came back, last year, we reckoned just having (nurse) available to us kept us out of hospital a hell of a lot just, even if, just having her there, just knowing that she's there means that when anything, you know that if, you've got that security if you want back up and you know that if anything does happen that you've got someone you can go to, or when something happens rather than kind of going 'what do we do?'

All of the children attended the local hospital and the specialist children's hospital for consultant review. Fathers commented on the support they receive from consultants in both settings. All of the children had "open access" to their local children's ward. This meant that if the child was unwell they could go directly to the ward and did not need referral from a General Practitioner (GP) or other healthcare professional. Two of the fathers talked about the good support they received from their GP and the regular contact they maintained. One of the children also accessed support from the Child and Adolescent Mental Health Service (CAMHS).

One father explained how they preferred all contact from professionals involved to be on their terms and to fit with the way they cared for their child. Father 4 talked about the support he had provided to other parents in their situation, and how important it was for professionals to find a balance and provide the appropriate level of support whilst at the same time respecting parents for the level of care they provide for their child:

'What we find is ourselves are usually the ones who are giving the advice out, and even your consultant, our consultant will tell us that we know better than they do on the management of the condition'.

When liaising with healthcare professionals on a regular basis fathers appreciated an awareness that they were the individuals involved in the day to day care and all aspects of their child's care.

Father 2 praised the services they received and felt that they would not have received this level of care elsewhere in the UK. He was offered a re-location package with work, but after an examination of the services in the relocation area decided to keep his family in the study area and travel a longer distance to work.

Overall, fathers felt involved in discussions with professionals that were aimed at both parents. For example, Father 5 felt parents were treated equally:

'I've not felt aware of anything that has specifically sort of alienated me because I'm a father if you see what I mean, I've not felt like there's any imbalance there at all, and especially at (hospital) anyway because you do get a lot of fathers staying at the hospital.....I can't really pinpoint anything that I thought that's happened to me because I'm a father'.

#### Experiences of negative challenges and fathers' needs not understood

Similar to most other studies predominantly with mothers, fathers had difficulty accessing appropriate social work support, and education input for their child.

One highlighted his need for respite. Father 7 explained:

'At the moment it's just respite. I mean that's the only thing we don't get at the moment is sort of you know someone to come in and give us just a couple of hours break.'

The general feeling expressed by fathers was that respite care did not always meet families' needs. The other fathers were either satisfied with provision or did not need respite.

### Fathers' experiences of support

The children being cared for required an enormous amount of extra care over and above those without complex healthcare needs [27]. The complexity of health, medical and nursing needs of these children meant that families could not adequately look after them without additional support from the State and Voluntary sector services. There is some evidence as to how parents of profoundly disabled children receive support (see for example [28]), and growing evidence about the lack of coordination and intrusion in family homes when children require complex and continuing care (see for example, [3,29]) but very little (if any) evidence as to how fathers of disabled children with complex healthcare needs perceive and receive either paid or voluntary support - especially if fathers are the main carer.

In the current study, support from extended family appeared limited mainly due to the complex needs of the child. All of the families were in varying degrees isolated either geographically or personally from their extended family and therefore relied on services for any additional support to care for their child.

Father 8 explained why this was the case:

'I'm from a ... certainly a decent sized family but we didn't really have the family support because, although sister's been good but I don't think they've understood, I really do not think they have understood what we have gone through'.

The fathers were aware that it might be difficult for family members to provide care due to the complexity of needs.

A number of the fathers received respite at home for their child. Respite care was provided during daytime hours or as overnight care if the child required complex nursing care during the night. Father 1 explained that although they were offered respite they preferred to care for their child themselves:

'It (respite) has been offered and I know we can sort of take it up but generally speaking it, you know I think we generally like to keep things as normal as possible'.

Ten years ago, a report from the Handsel Trust [30] highlighted that fathers of disabled children could be seen as 'Just a Shadow' within families, services and communities. A decade later with roles of fathers evolving to take on more aspects of complex health, medical and nursing care, the picture appears to have changed for the fathers in the current study, with fathers taking a prominent role and some finding it difficult to access support that met their particular needs as carers. Two of the fathers had attended support groups in the past but did not feel that they were aimed at fathers or met their needs. Both fathers had attended groups aimed at both parents but found they were too general therefore not aimed at their children. Also one father attended a group that only met quarterly and found this was too infrequent. Father 5 attended a number of children's groups with his son so he could integrate. He was often the only father, but continued to attend as he felt it was important to the child.

Only one father said he would find it useful meeting with other fathers. Father 6 stated:

'I mean to me if somebody set up some sort of...father thing, for fathers, and it was like a month or something I'd come along and meet people and that would be fine to me, you know, that would be worth, that'd be worth me attending because, like I say, you could access other people's information, and also discuss.....how you're feeling about your child'.

The CCN service organised yearly events, which most of the fathers did attend as part of family. Father 6 said that activities in the community were not always appropriate due to his child's complex needs and this lack of provision generally limited opportunities for a number of families.

Gaining support, talking and sharing their experiences outside of the family was valued by fathers in other studies [8,9,11]. Meeting with other parents through school or the hospital was of benefit to the fathers as they met with others in a similar situation to themselves. Father 3 had gained knowledge on appropriate benefits from another family:

'...that's right, but its, its as if, you actually have to know somebody, another parent, because where we learnt about this was at (name of hospital) oh you should be on this, you should be on that, I didn't know anything about that'.

In contrast, Father 8 preferred not to mix with other families.

Although some of the fathers may have valued meeting with other families this was not an easily available option. As identified earlier, fathers in this study already stressed that their spare time should be spent with their family. Healthcare professionals would need to work closely with fathers to plan an appropriate family-orientated fun event available to all, and possibly identify a crèche type facility where they could attend with their child. In the UK, the political landscape has also changed considerably in the last 12 months, and the economic recession is forcing hard funding decisions. The philosophy of the 'Big Society' has been coined and the expectation is that social support will largely be provided by families and communities and not the State. This community-based informal approach to family support warrants further exploration and analysis. Based on our findings this informal approach may 'fit' better with the way some fathers wish to manage their caring roles within their families, but it has yet to be seen if informal and voluntary support can be sustained over long periods of time.

#### Matching father's views with national policy context

When matching father's views and experiences against a policy backdrop, recent UK policies that for the first time guide implementation of children's ongoing complex healthcare say all the right things and share the same aspirations as fathers in this study. Every Child Matters [31], Children's NSF [5,32], Aiming High for Disabled Children [33], Improving the Life Chances of Disabled People [34], Better Care, Better Lives [35], and Children's Continuing Care National Service Framework [36], however, provide little guidance on the 'how to', that is implementation of appropriate father or parent and child-centred interventions at family and father level to ensure that children, fathers and mothers benefit from the resources, care and services provided by the State.

### Strengths and limitations

It is known to be challenging to recruit fathers to participate in qualitative studies and fathers are critically under represented in published studies on children and complex disability. The strategy used in the current study appeared to overcome some barriers to participation. Children's community nurses played a key role in sending or delivering by hand study information to home settings, and explaining about the study and what it involved in an individualised way to fathers. This personalised approach was invaluable and more successful in generating contact by fathers with the researchers, compared with techniques adopted in previous studies, whereby study information was sent through the post via the health service on our behalf and received unexpectedly with no follow-up from the service that sent it. The challenge however remains to overcome barriers to recruiting fathers who are not in contact with services, or in situations where health service staff are not able to provide a personalised approach to delivering and explaining study information.

This small study provided insight into the lives of 8 fathers, and we were most privileged to be able to report the subjective meanings of their experiences. Issues such as why fathers experience guilt at receiving support however warrant further exploration. The sample contained a wide age range of children and apart from the specific impacts of puberty and performing personal care, our study was not focussed on generating insights on the effects and impacts of age of children, or length of time the child required care on fathers' motives for, and experiences of, caring. We plan to investigate these aspects in subsequent studies.

Face to face interviews were also successful as a technique to elicit fathers' experiences. The female researcher was welcomed into family homes to meet fathers and their conversations were detailed and rich in data. In this study the female researcher (LH) has a high level of engaging communication skills and clinical nursing experience of working with fathers. She was very comfortable meeting and talking with fathers and it was clear from the digital recordings that fathers felt able to talk freely with her. Other less experienced professionals may need to consider what additional skills-based training they require to facilitate a similar level of positive engagement and rich data collection.

We adopted an analytical method which did not bracket our clinical experiences of working with children, fathers and families in home settings. We acknowledge that we cannot resolve the analytical differences between nurse/researchers and subject/fathers. As female nurse researchers, we cannot reconcile the roles of fathers or the responsibilities of parents.

We have however used our extensive experiences of the context of care and the complexities of caring for children with complex healthcare needs in family homes. This experience of living with and working alongside families in their homes has given specific and unique insight into the evolving roles of fathers and the challenges they face in developing their own caring roles for their children. We have used this rich contextual experience to understand the complexities of their situation, what they actually do in terms of complex caring, and to recognise and interpret meaning from what fathers do for their children.

We are not able to make specific judgments on whether a male researcher would have gained different types of information than a female researcher. In future, we will consider conducting further studies with male and female researchers to ascertain if fathers respond differently to male versus female gender or prefer different communication styles and personality types of the fieldworker.

## Conclusion

This study provides some of the first in-depth narratives from fathers who provide significant levels of complex nursing care for their disabled children with complex healthcare needs. Fathers were found to create or want to create structured routines that focussed on the father-child dyad when they were sole carers. Structured routines appeared to have differences to those organised and motivated by mothers when solely caring for their children. As more fathers adapt their roles to care for greater numbers of children with complex healthcare needs, it will become increasingly important to ensure that the psychological wellbeing of fathers as carers (as well as mothers) is understood.

This study also sets the scene for a future research agenda and raises many issues worthy of future exploration in a critically under researched and new area. Key areas of exploration include gaining a better understanding of fathers motivations and styles of caring, developing interventions to support fathers in their caring role, considering how best to support fathers to deliver intimate care to children and young people, and how to deliver tailored services to fathers in a family context when receipt of services and support appears to be associated with increased levels of guilt.

Family structures are increasingly complex and dynamic and it is common for disabled children to experience changes in family carers and caring roles over time. Given the flexible definition of 'father' commonly used in policy contexts, there needs to be greater understanding of the roles of men who take on the role of 'father' and their interplay within families where a child requires complex care. There is little understanding of fathering and caring by men who do not live at the family home, men who are themselves teenagers, or men who are step-parents etc. Finally, we need to learn more about the characteristics of fathers who have made a positive impact on their families, and despite the stresses have bucked the trend by developing strong and lasting relationships with their partners and disabled children. Knowing more about their resilience and coping may help inform interventions to support fathers who do not feel able to stay with their partner and parent a disabled child.

## Competing interests

The authors declare that they have no competing interests.

## Authors' contributions

LH and JN designed the study. LH collected data. LH and JN contributed to data analysis, interpretation of findings and drafting of the manuscript. Both authors read and approved the final manuscript.

## Pre-publication history

The pre-publication history for this paper can be accessed here:

http://www.biomedcentral.com/1472-6955/10/5/prepub
